# O017: A novel antiviral technology for air filtration

**DOI:** 10.1186/2047-2994-2-S1-O17

**Published:** 2013-06-20

**Authors:** T Pelet, F Matheux

**Affiliations:** 1 VIROBLOCK SA, Plan-les-Ouates, Switzerland

## Introduction

Bioaerosols exposure and potential impact on human health is a growing concern. Bioaerosols are assemblies of particles of variable biological origin (bacterial, viral, or fungal) suspended in the air and capable of initiating an infectious process in a susceptible host. They usually consist of a mixture of mono-dispersed and aggregate cells, spores, or viruses, carried by other materials, such as respiratory secretions. With rapid desiccation, the resultant smaller aerosols can remain airborne longer, while larger aerosols may initially fall out and then become re-suspended after desiccation.

## Objectives

Tests show that filter materials are very efficient at removing small particles (< 0.1 of um diameter) from air streams because electrostatic charge and Brownian motion trap these fine particles within the matrix of the fabric. Larger particles (>1.0um in diameter) such as bacteria or fungi are also efficiently removed by mechanical filtration. However, filtration media are generally poor at removing particles in the range from 0.1um to 0.5um in diameter, which are small enough to escape the forces of mechanical filtration yet are large enough to avoid being entrapped by electrostatic or Brownian motion. This “weakness window” unfortunately corresponds to the mean size of many viruses.

## Methods

Viroblock’s proprietary antiviral technology uses a combination of aliphatic lipidic chains, able to form lamellar structures, and specific cyclodextrins to inactivate enveloped viruses. The mode of action is based on cholesterol depletion from the viral membranes and, because cholesterol is present in most viral envelopes, the technology is active on all enveloped viruses tested so far. Importantly, when coated on non-woven fabrics, Viroblock’s technology retains its whole antiviral property.

## Results

Aerosol challenges were performed on a prototype antiviral facemask composed of a filtration layer associated to a Viroblock-coated external layer. Results show an overall reduction of more than five Log TCID_50_ (99.9997 %) of human Influenza virus H1N1.

## Conclusion

The broad spectrum activity coupled to the extreme rapidity of cholesterol depletion is opening the way to making antimicrobial fabrics that can inactivate air borne viruses during the very short time it takes for them to pass through a filter, hence providing an increased protection against bioaerosols of viral origin.

## Disclosure of interest

None declared.

